# Contributions of the voluntary local review process to policy integration: evidence from frontrunner cities

**DOI:** 10.1038/s42949-023-00101-4

**Published:** 2023-04-06

**Authors:** Fernando Ortiz-Moya, Marco Reggiani

**Affiliations:** 1 City Taskforce, Institute for Global Environmental Strategies. 2108-11, Kamiyamaguchi, Hayama Kanagawa, 240-0115 Japan; 2 University of Strathclyde, Department of Civil and Environmental Engineering, James Weir Building, Level 5, 75 Montrose Street, Glasgow, G1 1XJ Scotland UK

**Keywords:** Social policy, Development studies

## Abstract

The implementation of the Sustainable Development Goals (SDGs) relies on effective policy integration at all levels of government. However, integration across policy domains remains challenging for local authorities, particularly when it comes to articulating policies that recognise trade-offs and interactions between different SDGs. This study explores how the Voluntary Local Review (VLR) process—a tool to localise the 2030 Agenda—contributes to policy integration by thematically analysing interviews with city officials in 12 frontrunner cities that conducted a VLR between 2019 and 2020. Our results suggest three main ways in which the VLR process affects policy integration: (1) by facilitating cooperation and interdependencies between different policy sectors; (2) by creating new instruments to mainstream SDGs; and (3) by enhancing sustainability competencies. Hence, our study suggests that conducting a VLR has the transformative potential to achieve greater policy integration and further the 2030 Agenda.

## Introduction

The United Nations’ 2030 Agenda for Sustainable Development and its 17 Sustainable Development Goals (SDGs) outline a global agenda addressing humanity’s most pressing problems—ranging from climate change to preserving biodiversity and advancing gender equality^1^. Although the overall emphasis is on national governments^2^, there is increasing acceptance that the 2030 Agenda needs local implementation^3–6^. This is because local governments play a crucial role in delivering 105 out of the 169 targets comprised by the SDGs^7^. However, given that the SDGs were conceived with nation states in mind, local governments face substantial challenges in operationalising, implementing and monitoring the 2030 Agenda^8–10^. SDG localisation requires translating the SDGs into the local context, embedding their ethos into decision-making, financing their implementation, and developing a monitoring and follow-up framework with locally-adapted indicators^10,11^.

In addition to localisation, there is a growing consensus that successful SDG implementation relies on achieving policy integration^12,13^. More comprehensive cooperation across policy sectors is key to fostering interactions between SDGs in positive ways (synergies) while minimising negative ones (trade-offs)^10,14^—an aspect that is recognised in SDG 17.14, which calls to ‘enhance policy coherence for sustainable development’^1^. Whereas by linking policies sharing similar objectives to streamline efforts while limiting possible negative impacts on other policy domains^15–18^, policy integration helps to embed the three dimensions of sustainable development (i.e. economic, social and environmental) into governance structures and throughout all stages of policymaking.

However, operationalising policy integration is difficult. This is due, in part, to the ill-defined nature of the concept, which is often entangled in the literature with other ideas such as policy coherence or the nexus approach. Tosun and Leininger^18^ note that while ‘policy coherence’ is predominantly used by development studies scholars, ‘policy integration’ is commonly employed in environmental and climate change policy debates. Instead, the ‘nexus’ approach is focused on linking energy, food, water and climate change policy. Other obstacles to policy integration are related to the traditionally siloed nature of much local governance that defies the implicit interdependence of all the SDGs and their targets^19^. In contrast, integration between policy domains requires coordination of different departments from policy formulation to appraisal, the joint prioritisation of policy objectives^20^, and evidence-based mechanisms to identify interactions between goals and targets.

To overcome the challenges of SDG localisation and achieve policy integration, local and regional governments (LRGs) are increasingly resorting to Voluntary Local Reviews (VLRs)^21^. VLRs mirror in the local context the voluntary review and follow-up process of national governments, which are encouraged by the 2030 Agenda to produce Voluntary National Reviews (VNRs) at regular intervals to report their progress in achieving the SDGs^22,23^. Despite being a relatively new tool towards sustainable development, VLRs proved popular and by the end of 2021 almost 100 LRGs have presented one or more local reviews, the majority of them being municipal governments^4,24–26^. In addition, groups of LRGs are conducting their voluntary assessments of SDG implementation in the form of Voluntary Subnational Reviews (VSRs)^27^.

Unlike VNRs, VLRs lack official recognition within the review and follow-up architecture of the 2030 Agenda, and until recently, there was no formal definition of what a VLR is or how it should be conducted^21^. The first official UN-made guidelines around VLRs were published in 2020. The United Nations Economic and Social Commission for Asia and the Pacific (UNESCAP) guidelines draw on early VLRs to provide practical principles to facilitate local reviews tailored to its regional context^28^. The United Nations Department of Economic and Social Affairs (UNDESA) outlined elements for VLRs and VLR reports^29^. Also in 2020, the United Nations Human Settlements Programme (UN-Habitat) and the United Cities and Local Governments (UCLG) guidelines reviewed available VLR reports to identify typologies of local reviews, concluding with recommendations for future VLRs^4^. Meanwhile, the European Commission Joint Research Centre (EUJRC) released a handbook adapting the VLR process to Europe^30^, and the Institute for Global Environmental Strategies (IGES) presented a methodology to conduct VLRs based on the experience of Shimokawa, one of the first local governments in the world to present a VLR^31^. Afterwards, both the United Nations Economic Commission for Europe (UNECE) and the United Nations Economic Commission for Africa (UNECA) introduced their regional guidelines in 2021 and 2022^32,33^.

These guidelines broadly conceive a VLR as a process to assess LRGs’ progress towards the local implementation of the 2030 Agenda. They identify fundamental components within each phase of the local review process including aligning policy with the SDGs, engaging with stakeholders, collecting data, report writing, and creating follow-up and review frameworks. The importance of policy integration is usually noted in reference to synergies and trade-offs between the SDGs, or vertical integration between VNRs and VLRs. However, guidelines seldom clarify what is meant by integration or provide examples of how to operationalise integration through policymaking.

Based on the analysis of reports produced by frontrunner cities, prior research and official guidelines by international organisations highlight several benefits of VLRs. These include: (1) setting local priorities and policy alignment with the SDGs; (2) facilitating policy integration for sustainable development; (3) feeding into VNRs by providing on-the-ground data and localisation experiences; and (4) providing evidence-based monitoring tools^4,24,27,28,34^. While there is a growing body of literature exploring SDGs actions by local governments^35–38^, there is little empirical evidence to corroborate these claims—a situation that echoes the lack of evidence around the outcomes of policy integration processes put in place to address complex environmental issues^39,40^. Moreover, despite the emphasis on achieving policy integration when applying the SDGs, how to do so remains an understudied area of research, especially regarding organisational aspects and operationalisation^10,21,41,42^.

To address this gap, this paper explores VLRs as instruments for policy integration. Its main research question asks how the VLR process contributes to policy integration. We follow the models suggested by Turnpenny et al.^43^ and Tosun and Lang^44^ and assess this question across three key dimensions that are relevant in research on policy integration. These are: the motivations to conduct VLRs, the design of the VLR process, and the outcomes and the impacts on policymaking of VLRs. We investigate these issues through the thematic analysis of 11 semi-structured interviews and one open-ended questionnaire with city officers responsible for the VLR process from 12 frontrunner cities that conducted their local review between 2019 and 2020 (i.e. Barcelona, Bonn, Bristol, Buenos Aires, Espoo, Ghent, La Paz, Los Angeles, Pittsburgh, São Paulo, Taoyuan and Turku, see Supplementary Table 1 for a list of interview partners). The paper presents an in-depth analysis of the procedures and considerations followed by local governments when conducting local reviews, as well as the outputs and outcomes of VLRs beyond what is presented in the publicly available reports.

By focusing on the VLR process as a catalyst for greater policy integration, this paper contributes to research on the organisational and operational aspects of SDG localisation. As a result, the study aims to help the growing number of cities that are conducting local reviews in tackling the multidimensional challenges of sustainable development and policy integration more effectively.

The next section describes the key results from the thematic analysis. The paper then concludes by critically discussing the findings and providing recommendations for both scholars and practitioners.

## Results

### Conducting VLRs: external demands or policy integration

The analysis reveals that the motivation to conduct a VLR can be clustered into three themes: (1) an external drive influenced by forces outside of the local government; (2) an organisational drive to localise the SDGs at the city level; and (3) an explicit desire to foster policy integration in local policymaking thanks to the work done towards sustainable development. The degree to which each theme is relevant varies from city to city and contributes to shaping the overall VLR process.

External demands featured prominently in our interviews. Almost all participants highlighted that VLRs provided local governments with a unique chance to fulfil prior commitments on sustainable development or continue existing work on the SDGs encouraged by international organisations. Undertaking a VLR was a tangible way to account for the contributions made by each city to the 2030 Agenda and recognise the importance of this work on the global stage. These were welcomed opportunities given that, as noted by the interviewee from Los Angeles, for too long cities have been overlooked at international forums on sustainable development:

Sustainable development is not just advanced in multilateral spaces, where cities often don’t have a voice. Cities are continuously innovating in how service provision is delivered and programs are designed. … We’re always looking at how we [can] advance progress on the SDGs. … The VLR is a really important tool to help that [progress]. (I8)

If responding to increasing external demands from international organisations and city networks—such as UCLG and other UN Agencies—provided incentives, legitimacy and, in a few cases, resources to support the VLR process, there is little evidence that local governments were motivated by demands at the national level. In some instances, cities tried timing their VLR with their country’s VNR (like Bristol or Bonn) or were encouraged to start their review to align with a future and upcoming VNR (Taoyuan). However, this often ranked low in terms of motivation. The only notable exception is represented by Buenos Aires, where the VLR was facilitated by an agreement signed with the national government to commit to the localisation of the SDGs.

Local stakeholders were mentioned as another external force that was prominent in steering the VLR process. Although not always regarded as a primary motivation to start a VLR, stakeholders contributed to the review by providing additional support. Primary actors outside of municipal organisation ranged from existing SDGs networks (in Barcelona and Bristol), academia (Bristol and Pittsburgh), and citizens (Buenos Aires and Taoyuan) to philanthropic foundations (Los Angeles) or the third sector (São Paulo)—each bringing different perspectives and levels of engagement with SDGs localisation efforts.

Organisational drive varied in scope across all cities, reflecting differences in governance styles and planning cultures. Altogether, many interviewees reported that a key motivation behind the VLR was to operationalise the 2030 Agenda while providing a framework to monitor progress—a reasoning that aligns well with some of the most prominent external demands motivating the review process. As noted by the participant from Pittsburgh:

The VLR gave us a tool to [assess] what are the needs and gaps that we have in terms of advancing Sustainable Development Goals. … [It enabled us to assess] how city departments are providing services, policies or programmes that ultimately benefit the community. (I9)

In parallel, several cities conceived and used the local review as a communication tool to raise awareness of the SDGs among citizens and be transparent about their efforts toward sustainability. Thanks to VLRs, local governments could also seize the opportunity to join the growing international community of practice on SDG localisation and city networks fostering peer learning, as well as forging new partnerships to advance sustainable development.

In contrast, policy integration did not feature high among the concerns motivating VLRs—with only four interviewees hinting more explicitly at this issue. This is, in part, a surprising result as integration between existing policies seems to have implicitly underpinned the review processes in the cities included in this study (Table 1). A possible explanation for this outcome might be the emphasis placed by VLRs on monitoring and auditing policy instruments and programmes existing at the local level—even when policy integration is not amongst the explicit guiding principles of a VLR. As explained by Bonn’s interviewee, undertaking a VLR ‘brings together the broad picture’ of SDGs localisation and reveals ‘the interconnections between different policy fields’ (I2); thus it contributes to addressing issues of sustainability in a more integrated and effective way.

**Table 1:** Brief overview of the VLRs reports presented by the cities that were part of the study.

City	Region	Year(s)	Focus of VLR Report
Barcelona	Europe	2020	The VLR report overviews progress towards and highlights relevant strategies for all 17 SDGs. Barcelona selected 139 targets (out of the 169 comprised by the SDGs) and linked them to local indicators. The report concludes by exploring how the COVID-19 pandemic affected the localisation of the SDGs.
Bonn	Europe	2020	The VLR streamlines the 2030 Agenda into six municipal fields of action, and Bonn’s localisation focused on highlighting the interdependencies between different SDGs. To monitor progress, the VLR combined existing municipal indicators with benchmarks determined by the Association of German Cities and the Bertelsmann Foundation.
Bristol	Europe	2019	Bristol’s VLR followed the approval of the ‘Bristol One City Plan,’ which was conceived in accordance with the 2030 Agenda. The VLR report examines key trends in achieving the 17 SDGs, with data going back to 2010 (the chosen benchmark year) and concludes by highlighting the key challenges that emerged in conducting the local review.
Buenos Aires	LAC	2019, 2020	The 2019 VLR describes the process to institutionalise the 2030 Agenda and reviews the 6 SDGs recommended to nation states by the 2019 High-Level Political Forum. The 2020 VLR explores the challenges faced by the city and the localisation of the SDGs in the context of the COVID-19 pandemic.
Espoo	Europe	2020	The VLR is articulated into 3 sections: “Leave No One Behind,” “Let’s Do It Together,” and “Accelerate Action”. Together they compose the “Espoo Story”, a guiding strategy illustrating how the city is implementing the SDGs by collaborating with local stakeholders.
Ghent	Europe	2020	Ghent’s data-driven VLR presents in detail the city’s progress on the 2030 Agenda. Each SDG is assessed in an individual chapter outlining targets, indicators, and examples to illustrate concrete actions related to Ghent’s strategic development plan.
La Paz	LAC	2019	The VLR aligns the city’s masterplan with the 2030 Agenda while assessing progress towards implementing the SDGs. As a result, La Paz’s VLR identifies 3 priority areas for action including: ‘healthy life,’ ‘inclusive sustainable mobility,’ and ‘decent work.’
Los Angeles	North America	2019	Los Angeles’ VLR aims to raise awareness on the 2030 Agenda and presents a three-stages localisation strategy. First, it maps existing policies through the lenses of the SDGs. Second, the VLR identifies gaps and how to address them. Third, it identifies unique local priorities to monitor and accelerate progress.
Pittsburgh	North America	2020	The VLR extends the ongoing work on sustainable development initiated by the City of Pittsburgh Preliminary Resilience Assessment in 2016. On the one hand, it addresses fragmentation and helps to identify synergies and co-benefits between the city’s policies. The VLR, on the other hand, serves as a baseline report to understand the current state of implementation of the SDGs.
São Paulo	LAC	2020	São Paulo’s VLR describes organisations, partnerships, and strategies that exist in Brazil and São Paulo to promote sustainable development. The report explores current initiatives grouped into five themes: (1) institutional; (2) environmental; (3) economic; (4) social; and (5) fighting COVID-19 and recovery.
Taoyuan	Asia and the Pacific	2020	Taoyuan’s VLR mainstreamed the 5 Ps of sustainable development (People, Planet, Prosperity, Peace, and Partnership) across the city’s strategies. The VLR process encouraged municipal departments to review their ongoing operations. This resulted in a plan to implement the 2030 Agenda and the establishment of a review and follow-up framework.
Turku	Europe	2020	Turku’s VLR complements the city’s ongoing efforts on sustainable development—such as its commitment to be climate positive by 2029. By reviewing local initiatives against the SDGs, the report aligns the ‘Turku 2029 City Strategy’ with the 2030 Agenda. Through this exercise, Turku identified local indicators to track progress, as well as policy gaps and plans for addressing them.

Source: https://www.iges.or.jp/en/projects/vlr.

The table includes only VLRs published before data collection for the study (March–May 2021).

### The design of the VLR process

The design and implementation of the VLR process included substantial work within local governments. During the analysis, we identified two key themes that capture areas of particular concern for participants. These are: interdepartmental work and fostering stakeholder engagement. These two themes capture that city officers responsible for conducting the VLR usually required external support from other ‘siloes’ within the administration to grasp the breadth of ongoing strategies and the degree to which the SDGs were already embedded into policymaking. At the same time, they needed to collect relevant data to measure progress, which might be scattered across different departments and datasets. Similarly, VLR teams put a considerable amount of energy into engaging with local stakeholders to gather additional information and perspectives.

Cities designed different strategies to organise and operationalise interdepartmental work. Typically, city officers in charge of the VLR initiated mapping exercises to identify policy gaps and ways in which existing policies aligned with the SDGs, or they focused on facilitating conversations across departments. Other times, ad hoc VLR working groups were set up to oversee and streamline the local review process. As described by one of the participants from São Paulo:

We organised an internal group with all the main Secretariats of the city. … We tried to show the participants [in the VLR process] how the things they [had] already done and monitored could be used [as part of] our efforts to implement the SDGs …[and] to localise the 2030 Agenda. (I10)

Similarly, in Ghent, a task force was established to regularly report on the VLR process to the city’s coordination and management team.

Interviewees described how the VLR process intertwined with other local initiatives, predominantly those targeting cross-sectoral challenges such as climate action, gender, or racial and ethnic equity. For example, in Pittsburgh, the VLR was linked with the fossil fuel divestment strategy to achieve carbon neutrality by 2050. In Espoo, results from the local review led to reviewing the procurement process. To visualise the links across different targets and goals, the VLR team in Los Angeles promoted the creation of an open-source platform and an SDG Activities Index to capture all the SDG-related activities happening in the city and build shared knowledge.

To engage with stakeholders (which included citizens, the third sector, and a variety of other national and international organisations), city officers organised a wide range of surveys, workshops, public forums and meetings. These were instrumental in capturing stakeholders’ views on the review process and sometimes, as in the case of São Paulo, getting stakeholders’ input into designing and supporting the implementation of the VLR. While some cities were more successful than others in collaborating with external actors, all of them reported that public consultations made the local review process more transparent and democratic, added on-the-ground knowledge of citizens’ needs, and was key to their efforts in localising the SDGs. Some interviewees even suggested that, by engaging with the VLR, stakeholders gained a better understanding of the policymaking efforts towards sustainability in their cities and how they fit into those.

Both interdepartmental work and stakeholder engagement could be considered as means to achieve better policy integration. This is well exemplified by the case of Espoo, which stands out because of the effort made to explicitly embed integration into the design of its review process. Through the VLR, the city strove to incorporate the SDGs into key municipal processes such as budgeting or public relations. As such, Espoo sought to realise policy integration by bringing together the initiatives of its three largest departments (education and culture, health, and infrastructure), while helping internal capacity building and creating the conditions for better integration across policies in the future^45^ so that ‘everyone [in the organisation] can understand what their work means in relation to the [SDGs] targets and indicators’ (I5). Moreover, the city aimed at maximising engagement with citizens by working with students in middle and high schools, asking them what they considered the most relevant projects for the city—an activity that resulted in the selection of around 90 projects to be included in the VLR.

### The outcomes and impact on policymaking of the VLR process

Although it is too early to appreciate all the possible outcomes and the full impact of VLRs on policymaking, our analysis suggests that conducting local reviews forwarded different and complementary approaches to policy integration. The first pattern that we identified within the data relates to the ways VLRs advanced internal cooperation between different policy domains at the local government level in all the cities included in the study—an outcome that was often directly related to the design of the review process. Second, and in a more limited number of instances, during the VLR process new and specific instruments to mainstream and coordinate work on the SDGs were created. Finally, the local review process favoured an increase in the sustainability competencies of involved staff.

More effective cooperation across policy domains helped most of the municipal governments to assess, monitor, and connect actions of SDG localisation. As a result, the interdepartmental work undertaken during VLRs led to reformulating well-established policies in more coherent ways or creating new instruments that could reinforce municipal initiatives by finding synergies and trade-offs between policies. According to interviewees, policy domains in which this approach to policymaking was particularly beneficial include: procurement, mobility and transport infrastructures, education, gender, racial and ethnic equity, climate and energy, and disaster mitigation.

As highlighted by a number of cases, such as Turku and Bonn, VLRs were particularly efficient in facilitating the creation or the improvement of policy instruments to operationalise the measurement of progress towards the SDGs and set baselines for action—a finding that confirms the increased importance of appraisal in environmental policymaking^46^. Interviewees noted that VLRs helped to standardise and systematise evaluation tools (Barcelona and La Paz) and reporting frameworks (Bonn). They also helped to create online platforms to measure and report on local sustainability indicators (Los Angeles). These outputs represent one of the lasting legacies of VLRs since they ensure the continuity of the local review process and support evidence-based decisions in the future.

Another important outcome of the VLR process can be found in the increased sustainability competencies (SCs) of involved staff^47^—a theme which was recurrent in all the interviews. Wiek et al. define sustainability competencies as the ‘complexes of knowledge, skills, and attitudes that enable successful task performance and problem-solving concerning real-world sustainability problems, challenges, and opportunities’^48^. Although originated in debates concerning education and curriculum development, SCs have recently received growing attention in relation to local governments^49,50^. In particular, interviewees reported that while engaging in the VLR, staff acquired key components of SCs, i.e. ‘systems-thinking, futures-thinking, values-thinking, strategic-thinking, and interpersonal competencies’^51^. The ability of VLRs to increase SCs was perceived as key to empowering individuals and organisations to address complex challenges (e.g. climate change), as captured during the conversation with the participant from Turku:

[Our VLR] is a new framework, [and also] a new language. … Cities already do very much in relation to [the SDGs]. But [the VLR provides] a new lens through which we can actually see … [how] everyday tasks and activities are linked. I think it’s a very useful tool to … creating [a new] understanding. (I11).

Increased SCs were achieved in two interrelated ways. On the one hand, conducting VLRs provided a better understanding of the SDGs and their embedded synergies and trade-offs; for example, by identifying future goals with measurable targets and reflecting on how to recalibrate municipal operations based on the principles of the 2030 Agenda. On the other hand, by adopting participatory approaches (either within the local administration or with external stakeholders, or both), engaging with the review process contributed to greater communication and interpersonal skills—which have all been discussed as key competencies linked to SCs^49^. As Pittsburgh’s interviewee remarked: ‘[the VLR] allowed us to provide a … comprehensive and introspective view with regards to how the SDGs affect city government services’ (I9). While it is hard to measure the impact of this capacity-building exercise on the policymaking process, if sustained in the future this approach will arguably create an institutional environment that is more conducive to policy integration.

## Discussion

This paper examines how the process of conducting a Voluntary Local Review (VLR), which is becoming popular as an instrument to accelerate the localisation of the SDGs^21,24–26^, contributes to policy integration in local policymaking. We approached this question through the thematic analysis of 11 semi-structured interviews and one open-ended questionnaire with city officers responsible for guiding and conducting the local review process in 12 cities that completed their VLR between 2019 and 2020. The themes we generated during the analysis were organised and presented within three overarching topics that have been identified in the literature as key lines of inquiry when it comes to research on policy integration—i.e. (1) motivation; (2) design; and (3) outcomes and impact^43,44^.

Findings suggest that even though VLRs were usually not conceived as tools to foster policy integration, as demonstrated by the low number of interviewees who mentioned this theme as a motivation to undertake a VLR, the design and the implementation of the local review processes resulted in both organisational and operational steps conducive to better coordination across policy domains. This may be because the VLR process encourages city officials to reflect on the benefits of integrated policymaking and the interdependent nature of the SDGs when planning for their localisation. Since our cases were all early adopters of the VLR process, on the other hand, better levels of policy integration obtained after conducting VLRs might be explained by considering that these cities were already invested in achieving greater integration to advance sustainable development. While more comparative research would be needed to understand the circumstances under which cities decide to submit VLRs, it seems sensible to suggest that both the format of the VLR process and cities’ ongoing commitments to the 2030 Agenda play a role in determining better policy integration over time.

Our analysis reveals three ways in which the VLR process contributes to policy integration: (1) by facilitating cooperation and interdependencies between different sectors in local governments; (2) by creating new and specific instruments to mainstream work on the SDGs; and (3) by increasing the sustainability competencies (SCs) of municipal staff. However, with few exceptions, there was not much concrete impact on governance structures—something that, it is worth noting, may have still occurred after our interviews. Apart from the fact that furthering policy integration did not feature high among the motivations to undertake VLRs, this result might be explained by the lack of an explicit mandate to significantly transform governance structures through local reviews.

This paper highlights that VLRs helped cities in establishing interdependencies between different policy domains by acting as procedural policy instruments^52^. This is because VLRs were used as frameworks to achieve coordinated thinking between departments^53^ and to structure policy appraisal^20,43,46,54^. In a few instances, new interactions between policy domains resulted in the revision of core municipal processes, such as public procurement. By identifying appropriate coordination mechanisms, VLRs also concurred to eliminate redundancies and incoherencies^55–57^. These outcomes align with the central tenets of policy integration, particularly when it comes to moving past traditional siloed approaches in local government, thereby realising holistic solutions to simultaneously advance several SDGs.

As our analysis shows, VLR’s teams and local governments usually resort to the creation of ad hoc working groups or special committees to work across departments. This approach has both strengths and weaknesses. On the one hand, it allows people to gather quickly and with a strong mandate to work on a specific goal, e.g. SDGs localisation. On the other hand, without being adequately embedded in government structures, the scope of VLR working groups remains limited and heavily dependent on political cycles—which are usually much shorter than the time needed to substantially advance integrated approaches to policymaking and sustainable development^58^—or external resources—since funding is critical to implementing sustainability-related policies^59^. This lessens the impact of VLRs on policy integration since the review might be undertaken as a one-time institutional exercise rather than becoming a blueprint for a more sustainable and integrated approach to policymaking that is firmly established at the core of political systems and institutional practices.

As expected, VLRs led to the creation of specific instruments to monitor and evaluate municipal actions against the 2030 Agenda—which, similar to what happens with other initiatives of environmental policy integration^60^, acted as a powerful incentive on the political motivations that exist in the background of VLRs. Both Barcelona and Los Angeles created publicly available SDG monitoring tools based on the Open SDG platform—an open-source and free-to-reuse tool for publishing data relevant to SDG indicators^61,62^. By presenting up-to-date data in an accessible manner, these platforms keep cities accountable to their citizens and relevant stakeholders. This in turn can support policy integration by allowing for a comparison of indicators or identification of trade-offs and interlinkages between goals and targets^63^. At the local level, these tools can be used for follow-up VLRs and to deepen the understanding of the SDGs. Whereas at a larger scale they might inspire other cities to implement similar platforms when conducting their VLRs—which would be beneficial to compare indicators and outcomes both across and within countries.

A notable finding of this paper is that VLRs contributed to enhancing the SCs of municipal staff. This seemed to be a rather unintended consequence of the local review process that happened organically as the work evolved, by providing training opportunities for staff on issues related to sustainability and the SDGs. As a result, by engaging with the VLR process, staff became aware of the interactions between policy domains and how to better coordinate to achieve goals and targets. These are skills that, albeit more difficult to measure, might be beneficial to policy integration because lack of capacity—not limited to resources but including skills and knowledge—has been recognised as a key barrier to achieving better synergies in policymaking^64,65^.

The participatory setting of VLRs, which facilitates knowledge exchange between local governments and stakeholders, might further enhance the SCs of all actors involved —thus increasing momentum toward the localisation of the SDGs and supporting the idea that policy integration, when applied to environmental issues, can be regarded as a ‘learning moment’^40,60^. It is noteworthy that the ability of VLRs to foster SCs might be particularly beneficial for cities that are behind in their SDG localisation journey. This is because conducting a local review gives municipal staff the chance to proactively create connections and opportunities to delivering the 2030 Agenda both inside and outside local governments, which might eventually help to mitigate and overcome adverse institutional contexts.

Our study reveals that, despite benefits on procedures and organisational aspects of governance, the policy integration brought by VLRs had a more limited impact on the substantial restructuring of ingrained management or policymaking paradigms. This finding aligns with Jordan and Lenschow^40^ who, in their review, highlighted the difficulty of transforming overarching government principles and strategies as one of the common shortcomings of policy integration exercises. Another possible explanation for these results is that most of the participants undertook their VLR as a truly bottom-up exercise—i.e. with limited guidelines or models to follow, they had to find their own ways to engage with the 2030 Agenda. Moreover, our interviews comprised early adopters of VLRs. Hence, the main focus of those involved in the review process was on reporting progress on the SDGs with advancements in policy integration resulting as by-products.

While we agree with recent research indicating that the 2030 Agenda seems to have had limited and largely discursive impact on both local and global governance so far^66^, our study indicates that, overall, conducting a VLR is beneficial to advance policy integration. The empirical evidence analysed and discussed in this paper contributes to better understanding of the transformative potential of VLR processes. By identifying and discussing challenges and inconsistencies of the review process, as well as successful initiatives and best practices from frontrunner cities, we found that VLRs contribute to bridging gaps across policy domains, mainstreaming procedures and operations, and embedding principles of integration and sustainability in policymaking—which are all key to SDG localisation^12,13^. This constitutes valuable insight to inspire the next generation of VLRs and, more in general terms, to help local governments to achieve the SDGs.

It is still not possible to fully grasp and evaluate all the potential ways in which the VLR process might contribute to policy integration. Although four of our cases had conducted their VLR in 2019, the majority of VLRs dated from 2020, and our interviews took place in early 2021. Therefore, it is likely that some outcomes from the VLR process that are related to policy integration have yet to appear. This warrants further and cross-disciplinary investigation. Areas that might benefit from future research include institutional and political aspects of VLR implementation, the ways in which VLRs articulate the localisation of global agendas, measuring and comparing the outcomes and impact of VLRs across policy domains, or exploring how the VLR process can contribute to issues of SCs. To local governments undertaking VLRs, we would recommend considering policy integration issues from the very beginning of the review process to maximise benefits and commit adequate resources, funding and staffing to build capacity around sustainability. VLRs can and should help further the 2030 Agenda. But without fundamental shifts in policymaking, little of substance can be achieved when it comes to ensuring a more sustainable future.

## Methods

### Cases and participants

For this study, we aimed to include a sample as complete and diverse as possible of the cities that conducted a VLR between 2019 and 2020. In that period a total of 40 cities around the world had completed and published a VLR. By following the statistical regions defined by the United Nations Statistics Division, but dividing the Americas into North America and Latin America and the Caribbean for greater clarity, the VLRs presented by 2020 correspond to cities distributed among the following regions: 14 in Europe, followed by 13 in Latin America and the Caribbean Region, seven in Asia and the Pacific, four in Africa, and three in North America. More than 20 cities were contacted to scope their interest and availability in participating in the study. Options were given to participate in an interview or submit their reflection through an open-ended questionnaire.

Out of those contacted, representatives from 12 cities agreed to join the study (see Table 1 for a brief overview of the VLRs reports presented by the cities that were part of the study). Four cities were early adopters of the VLR format (Bristol, Buenos Aires, La Paz and Los Angeles) since they conducted their local review in 2019; the remaining nine cities presented their first VLR in 2020. At the time of the interview, Buenos Aires had already carried out its second VLR. Roughly half of the case are located in Europe (Barcelona, Bonn, Bristol, Espoo, Ghent and Turku), two in North America (Los Angeles and Pittsburgh), three in Latin America and the Caribbean (Buenos Aires, La Paz and São Paulo), and one in Asia and the Pacific (Taoyuan). Cities in Africa were also contacted but did not respond. Whilst the absence of cases from the African continent represents a limitation, the inclusion of more than one-quarter of the cities that conduct a VLR within the time frame considered by the study resulted in reaching a sufficient saturation^67^.

A total of 14 participants were recruited by the corresponding author (FOM) by using both existing contacts and establishing new connections with representatives in each of the cases. We sought to recruit city officers who were responsible for the VLR process This choice was made to better understand the inner functioning of the local review process since participants were directly involved in guiding and developing the VLR of their respective cities.

### Data

Eleven semi-structured interviews and one open-ended questionnaire were conducted between March and June 2021. The aim was to broadly understand motivations, design and implementation, along with outcomes and impact of the VLR process in each of the cases. To ensure that all the relevant information was collected, a protocol was developed as a guide for interviews. Key questions included: Why did your city decide to conduct a VLR?; What was the main incentive?; How is the VLR integrating with other existing policies?; and, How is the VLR process influencing governance structures? During and after interviews the protocol was revised to adapt to the natural flow of the conversation and focus on those questions that were found to elicit better insight from participants.

Interviews were conducted and recorded online by the corresponding author (FOM). Ethical approval for the research was obtained by the corresponding author (FOM) from the Institute for Global Environmental Strategies (IGES), Japan and consent to take part in the study was obtained from participants. To protect confidentiality, quotes included in the paper have been anonymised.

### Coding and thematic analysis

Thematic analysis^68,69^ was used to explore and identify themes within the data. To code the data, we employed a combination of deductive and indicative approaches. This methodology has been found to be both systematic and flexible, and more representative of the process of coding and analysis actually employed by most researchers^70,71^. As such, coding was both theory and data-driven to structure the analysis around issues of policy integration recognised in the literature (i.e. motivation, design, outcomes and impact) without sacrificing the rich variety of information included in the interviews.

After transcribing the interviews, and before the systematic analysis of data, a codebook was developed by the authors that included three broad codes (the motivations to conduct VLRs, the design of the VLR process, and the outcomes and the impacts on policymaking of VLRs). Tentative subcodes were defined and added to the codebook in the preliminary stage of the analysis to further organise and clarify the links within the data. For each code, we provided a label, a working definition, and a succinct outline describing when and how some codes were likely to occur in the conversation. By employing an interactive and reflexive approach to coding, we left room to adjust, reorganise and clarify codes. A sample of representative interviews was coded independently by the authors, who then compared coding choices. Multiple rounds of preliminary coding were conducted to improve the trustworthiness and reliability of the analysis. During this process, themes started to be identified and the process was repeated until agreement was reached between the authors.

## Supplementary information

Supplementary table 1

## Data Availability

The data are not publicly available as they contain information that could compromise the privacy/consent of research participants. Explicit consent to deposit raw transcribed data was not obtained from participants. Upon reasonable and valid request, the data that support the findings of this study are available from the corresponding author [FOM].

## References

[1] UNGA, (United Nations General Assembly). *Transforming our world: the 2030 Agenda for Sustainable Development*. (2015).

[2] Graute U (2016). Local authorities acting globally for sustainable development. Reg. Stud..

[3] Jones P, Comfort D (2020). A commentary on the localisation of the sustainable development goals. J. Public Aff..

[4] UCLG & UN-Habitat. *Guidelines for voluntary local reviews: Vol. 1, A Comparative Analysis of Existing VLRs*. (UCLG and UN-Habitat, 2020).

[5] Immler NL, Sakkers H (2022). The UN-Sustainable Development Goals going local: learning from localising human rights. J. Hum. Rights.

[6] UN General Assembly. *Political declaration of the high-level political forum on sustainable development convened under the auspices of the General Assembly*. (2019).

[7] OECD. *A territorial approach to the sustainable development goals: Synthesis report.* (OECD Publishing, 2020).

[8] Rudd, A., Simon, D., Cardama, M., Birch, E. L. & Revi, A. The UN, the Urban sustainable547development goal, and the new urban agenda. in urban planet: knowledge towards sustainable cities548(eds. Elmqvist, T. et al.) 180–196 (Cambridge University Press, 2018).

[9] Sachs JD (2019). Six transformations to achieve the sustainable development goals. Nat. Sustain..

[10] Stafford-Smith M (2018). Advancing sustainability science for the SDGs. Sustain. Sci..

[11] Tan DT (2019). Systems approaches for localising the SDGs: co-production of place-based case studies. Glob. Health.

[12] Biermann F, Kanie N, Kim RE (2017). Global governance by goal-setting: the novel approach of the UN sustainable development goals. Curr. Opin. Environ. Sustain..

[13] Breuer A, Janetschek H, Malerba D (2019). Translating sustainable development goal (SDG) interdependencies into policy advice. Sustainability.

[14] Brand A, Furness M, Keijzer N (2021). Promoting policy coherence within the 2030 agenda framework: externalities, trade-offs and politics. Politics Gov.

[15] Mickwitz, P. et al. *Climate policy integration, coherence and governance*. (Partnership for European Environmental Research, 2009).

[16] Coscieme L, Mortensen LF, Donohue I (2021). Enhance environmental policy coherence to meet the sustainable development goals. J. Clean. Prod..

[17] OECD. *Better policies for sustainable development 2016: a new framework for policy coherence*. (OECD Publishing, 2016).

[18] Tosun J, Leininger J (2017). Governing the interlinkages between the sustainable development goals: approaches to attain policy integration. Global Challenges.

[19] Nilsson M, Griggs D, Visbeck M (2016). Policy: map the interactions between sustainable development goals. Nature.

[20] Russel D, Jordan A (2009). Joining up or pulling apart? the use of appraisal to coordinate policy making for sustainable development. Environ. Plan. A.

[21] Narang Suri S, Miraglia M, Ferrannini A (2021). Voluntary local reviews as drivers for SDG localisation and sustainable human development. J. Hum. Dev. Capab..

[22] Elder, M. *Assessment of ASEAN countries’ concrete SDG implementation efforts: policies and budgets reported in their 2016–2020 voluntary national reviews (VNRs)*. (Institute for Global Environmental Strategies, 2020).

[23] Elder, M. & Bartalini, A. *Assessment of the G20 countries’ concrete SDG implementation efforts: policies and budgets reported in their 2016–2018 voluntary national reviews*. (Institute for Global Environmental Strategies, 2019).

[24] Ciambra, A. & Martinez, R. *Voluntary local reviews, VLRs toolbox: from data analysis to citizen engagement when monitoring the SDGs*. (UCLG, UN-Habitat, and ICLD, 2022).

[25] Ortiz-Moya, F., Saraff Marcos, E., Kataoka, Y. & Fujino, J. *State of the voluntary local reviews 2021: from reporting to action*. (Institute for Global Environmental Strategies, 2021).

[26] Ortiz-Moya, F. & Kataoka, Y. *State of the voluntary local reviews 2022: overcoming barriers to implementation*. (Institute for Global Environmental Strategies, 2022).

[27] UCLG & UN-Habitat. *Guidelines for voluntary local reviews: Vol. 2, towards a new generation of VLRs: exploring the local-national link*. (UCLG and UN-Habitat, 2021).

[28] UNESCAP. *Asia-Pacific regional guidelines on voluntary local reviews: reviewing local progress to accelerate action for the sustainable development goals*. (United Nations Economic and Social Commission for Asia and the Pacific, 2020).

[29] UNDESA. *Global guiding elements for voluntary local reviews (VLRs) of SDG implementation*. (United Nations Department for Economic and Social Affairs, 2020).

[30] Siragusa, A., Vizacaino, P., Proietti, P. & Lavalle, C. *European handbook for SDG voluntary local reviews*. (European Commission, Joint Research Centre, 2020).

[31] Koike, H., Ortiz-Moya, F., Kataoka, Y. & Fujino, J. *The shimokawa method for voluntary local review (VLR), a blueprint to localise the sustainable development goals*. (Institute for Global Environmental Strategies, 2020).

[32] UNECA. *Africa voluntary local review guidelines*. (United Nations Economic Commission for Africa, UCLG Africa, and UN-Habitat, 2022).

[33] UNECE. *Guidelines for the development of voluntary local reviews in the ECE region*. (United Nations Economic Commission for Europe, 2021).

[34] Ortiz-Moya, F., Koike, H., Ota, J., Kataoka, Y. & Fujino, J. *State of the voluntary local reviews 2020: local action for global impact in achieving the SDGs*. (Institute for Global Environmental Strategies, 2020).

[35] Jiménez-Aceituno A, Peterson GD, Norström AV, Wong GY, Downing AS (2020). Local lens for SDG implementation: lessons from bottom-up approaches in Africa. Sustain. Sci..

[36] Leavesley A, Trundle A, Oke C (2022). Cities and the SDGs: Realities and possibilities of local engagement in global frameworks. Ambio.

[37] Ozawa-Meida L (2021). Integrating the sustainable development goals (SDGs) into urban climate plans in the UK and Japan: a text. Analysis. Climate.

[38] Zinkernagel R, Evans J, Neij L (2018). Applying the SDGs to cities: business as usual or a new dawn?. Sustainability.

[39] Biesbroek R (2021). Policy integration and climate change adaptation. Curr. Opin. Environ. Sustain..

[40] Jordan A, Lenschow A (2010). Environmental policy integration: a state of the art review. Env. Pol. Gov..

[41] Masuda, H. et al. SDGs mainstreaming at the local level: case studies from Japan. *Sustain. Sci*. 1539–1562 (2021).

[42] Horan D (2020). Enabling integrated policymaking with the sustainable development goals: an application to Ireland. Sustainability.

[43] Turnpenny J, Radaelli CM, Jordan A, Jacob K (2009). The policy and politics of policy appraisal: emerging trends and new directions. J. Eur. Public Policy.

[44] Tosun J, Lang A (2017). Policy integration: mapping the different concepts. Policy Stud.

[45] Taajamaa V, Joensuu M, Karanian B, Bettencourt L (2022). Seven steps to strategic SDG sensemaking for cities. Adm. Sci..

[46] Owens S, Rayner T, Bina O (2004). New agendas for appraisal: reflections on theory, practice, and research. Environ. Plan. A.

[47] Perez Salgado F, Abbott D, Wilson G (2018). Dimensions of professional competences for interventions towards sustainability. Sustain. Sci..

[48] Wiek A, Withycombe L, Redman CL (2011). Key competencies in sustainability: a reference framework for academic program development. Sustain. Sci..

[49] MacDonald A, Clarke A, Ordonez-Ponce E, Chai Z, Andreasen J (2020). Sustainability managers: the job roles and competencies of building sustainable cities and communities. Public Perform. Manag. Rev..

[50] Zeemering ES (2018). Sustainability management, strategy and reform in local government. Public Manag. Rev..

[51] Redman A, Wiek A, Barth M (2021). Current practice of assessing students’ sustainability competencies: a review of tools. Sustain. Sci..

[52] Peters, B. G. *Pursuing horizontal management: the politics of public sector coordination*. (University Press of Kansas, 2015).

[53] Pollitt C (2003). Joined-up government: a survey. Political Stud. Rev..

[54] Yonehara A (2017). The role of evaluation in achieving the SDGs. Sustain. Sci..

[55] Bolleyer N (2011). The influence of political parties on policy coordination: political parties and policy coordination. Governance.

[56] Peters BG (1998). Managing horizontal government: the politics of co-ordination. Public Adm..

[57] Peters, B. G. Concepts and theories of horizontal policy management. In *Handbook of public policy* (eds. Peters, B. G. & Pierre, J.) vol. 1 115–138 (SAGE Publications, 2006).

[58] Dovers SR, Handmer JW (1993). Contradictions in sustainability. Environ. Conserv..

[59] Bulkeley H, Kern K (2006). Local government and the governing of climate change in Germany and the UK. Urban Stud..

[60] Nilsson M, Persson A (2003). Framework for analysing environmental policy integration. J. Environ. Policy Plan..

[61] Barcelona City Council. *Annual monitoring and evaluation report on the Barcelona 2030 Agenda (Voluntary Local Review 2021*). (2021).

[62] City of Los Angeles. *Los Angeles Sustainable Development Goals: 2021 Voluntary Local Review of Progress toward the Sustainable Development Goals in Los Angeles*. (2021).

[63] Karlsson-Vinkhuyzen S, Dahl AL, Persson Å (2018). The emerging accountability regimes for the sustainable development goals and policy integration: friend or foe? *Environ. Plan C Politics*. Space.

[64] Hertin J, Berkhout F (2003). Analysing institutional strategies for environmental policy integration: the case of EU enterprise policy. J. Environ. Policy Plan..

[65] Ross A, Dovers S (2008). Making the harder yards: environmental policy integration in Australia. Aust. J. Public Adm..

[66] Biermann F (2022). Scientific evidence on the political impact of the sustainable development goals. Nat. Sustain..

[67] Saunders B (2018). Saturation in qualitative research: exploring its conceptualization and operationalization. Qual. Quant..

[68] Braun, V. & Clarke, V. *Thematic analysis: a practical guide*. (SAGE Publications Ltd, 2021).

[69] Guest, G., MacQueen, K. M. & Namey, E. E. *Applied thematic analysis*. (Sage Publications, 2012).

[70] Braun, V. & Clarke, V. Thematic analysis. In *APA handbook of research methods in psychology, Vol. 2: Research designs: Quantitative, qualitative, neuropsychological, and biological*. (eds. Cooper, H. et al.) 57–71 (American Psychological Association, 2012).

[71] Swain, J. *A Hybrid Approach to Thematic Analysis in Qualitative Research: Using a practical example*. (SAGE Publications, 2018).

