# Supplementary material for: Contributions of the voluntary local review process to policy integration: evidence from frontrunner cities
Source: NPJ Urban Sustain. 2023 Apr 6;3(1):22. doi: 10.1038/s42949-023-00101-4 (PMC10078030; doi:10.1038/s42949-023-00101-4)
Supplement: Supplementary file 1 — Supplementary table 1 [file 42949_2023_101_MOESM1_ESM.docx]

**Supplementary Table 1: List of Interviewed Cities**

| **Interview** | **Cases** | **Organisation** | **Date** |
| --- | --- | --- | --- |
| Interview 1 | Barcelona | City of Barcelona | 23.03.2021 |
| Interview 2 | Bonn | City of Bonn | 25.03.2021 |
| Interview 3 | Bristol | City of Bristol | 20.04.2021 |
| Interview 4 | Buenos Aires | City of Buenos Aires | 06.04.2021 |
| Interview 5 | Espoo | City of Espoo | 01.04.2021 |
| Interview 6 | Ghent | City of Ghent | 01.04.2021 |
| Interview 7 | La Paz | City of La Paz | 09.06.2021 |
| Interview 8 | Los Angeles | City of Los Angeles | 31.03.2021 |
| Interview 9 | Pittsburgh | City of Pittsburgh | 27.04.2021 |
| Interview 10 | São Paulo | City of São Paulo | 12.05.2021 |
| Interview 11 | Turku | City of Turku | 29.03.2021 |
| Questionnaire 1 | Taoyuan | City of Taoyuan | 12.05.2021 |
